# Relationship between pre-extubation positive endexpiratory pressure and oxygenation after coronary artery bypass grafting

**DOI:** 10.5935/1678-9741.20150044

**Published:** 2015

**Authors:** Reijane Oliveira Lima, Daniel Lago Borges, Marina de Albuquerque Gonçalves Costa, Thiago Eduardo Pereira Baldez, Mayara Gabrielle Barbosa e Silva, Felipe André Silva Sousa, Milena de Oliveira Soares, Jivago Gentil Moreira Pinto

**Affiliations:** 1 Hospital Universitário da Universidade Federal do Maranhão (HUUFMA), São Luís, MA, Brazil.; 2 Universidade Estadual do Piauí (UESPI), Teresina, PI, Brazil.

**Keywords:** Oxygenation, Positive-Pressure Respiration, Intrinsic, Coronary Artery Bypass

## Abstract

**Introduction:**

After removal of endotracheal tube and artificial ventilation, ventilatory support should be continued, offering oxygen supply to ensure an arterial oxygen saturation close to physiological.

**Objective:**

The aim of this study was to investigate the effects of positive-end expiratory pressure before extubation on the oxygenation indices of patients undergoing coronary artery bypass grafting.

**Methods:**

A randomized clinical trial with seventy-eight patients undergoing coronary artery bypass grafting divided into three groups and ventilated with different positive-end expiratory pressure levels prior to extubation: Group A, 5 cmH_2_O (n=32); Group B, 8 cmH_2_O (n=26); and Group C, 10 cmH_2_O (n=20). Oxygenation index data were obtained from arterial blood gas samples collected at 1, 3, and 6 h after extubation. Patients with chronic pulmonary disease and those who underwent off-pump, emergency, or combined surgeries were excluded. For statistical analysis, we used Shapiro-Wilk, G, Kruskal-Wallis, and analysis of variance tests and set the level of significance at *P*<0.05.

**Results:**

Groups were homogenous with regard to demographic, clinical, and surgical variables. There were no statistically significant differences between groups in the first 6 h after extubation with regard to oxygenation indices and oxygen therapy utilization.

**Conclusion:**

In this sample of patients undergoing coronary artery bypass grafting, the use of different positive-end expiratory pressure levels before extubation did not affect gas exchange or oxygen therapy utilization in the first 6 h after endotracheal tube removal.

## Abstract (pt)

**Introdução:**

Após a remoção do tubo endotraqueal e ventilação artificial, o suporte ventilatório deve ser continuado, oferecendo suprimento de oxigênio para garantir uma saturação arterial de oxigênio próxima da fisiológica.

**Objetivo:**

O objetivo deste estudo foi investigar os efeitos da pressão expiratória positiva final antes de extubação nos índices de oxigenação de pacientes submetidos à cirurgia de revascularização miocárdica.

**Métodos:**

Ensaio clínico randomizado com 78 pacientes submetidos à cirurgia de revascularização do miocárdio, divididos em três grupos e ventilados com diferentes níveis de pressão expiratória positiva final antes da extubação: Grupo A, 5 cmH_2_O (n=32); Grupo B, 8 cm H_2_O (n=26); e grupo C, 10 cmH_2_O (n=20). Dados do índice de oxigenação foram obtidos a partir de amostras de gases sanguíneos arteriais coletados em 1, 3 e 6 h após a extubação. Pacientes com doença pulmonar crônica e aqueles que foram submetidos à cirurgia sem circulação extracorpórea, de emergência ou combinadas foram excluídos. Para a análise estatística, foram utilizados Shapiro-Wilk, G, Kruskal-Wallis, e análise dos testes de variância e definição do nível de significância em *P*<0,05.

**Resultados:**

Os grupos foram homogêneos em relação às variáveis ​​demográficas, clínicas e cirúrgicas. Não houve diferenças estatisticamente significativas entre os grupos nas primeiras 6 h após extubação no que diz respeito aos índices de oxigenação e a utilização de oxigenoterapia.

**Conclusão:**

Nesta amostra de pacientes submetidos à revascularização do miocárdio, o uso de diferentes níveis de pressão expiratória positiva final antes da extubação não afetou as trocas gasosas ou utilização de oxigenoterapia nas primeiras 6h após a remoção do tubo endotraqueal.

**Table:** 

**Abbreviations, acronyms & symbols**	
BMI	Body mass index
CABG	Coronary artery bypass grafting
FiO_2_	Inspired oxygen fraction
FiO_2_N	Necessary inspired oxygen fraction
ICU	Intensive Care Unit
IMV	Invasive mechanical ventilation
NIV	Noninvasive ventilation
PaO_2_	Arterial oxygen partial pressure
PaO_2_I	Ideal arterial oxygen partial pressure
PEEP	Positive end-expiratory pressure
PSV	Pressure support ventilation
SaO_2_	Arterial oxygen saturation
SBT	Spontaneous breathing trial

## INTRODUCTION

Coronary artery bypass grafting (CABG) is a therapeutic modality widely used to treat coronary artery disease, minimize symptoms, improve cardiac function, and improve survival^[1,2]^.

Intraoperative conditions, such as general anesthesia, manual compression of the left lower lung lobe during exposure of the posterior heart surface, manual compression of the right lung during cannulation of the inferior vena cava, manual compression of lungs during dissection of the internal mammary artery and apnea during cardiopulmonary bypass (CPB) may impair pulmonary function^[3]^. Thus, pulmonary complications occur in up to 60% patients undergoing CABG^[4]^.

Invasive mechanical ventilation (IMV) is essential during the first few hours after CABG to allow recovery from anesthesia and reestablish homeostasis^[5]^. Typical restoration of hemodynamic stability occurs 5–6 h after surgery in uncomplicated CABG. This interval also correlates with regaining of consciousness and IMV weaning^[6]^.

When IMV is no longer required, the most appropriate method for its discontinuation must be determined^[7]^. The spontaneous breathing trial (SBT) is a simple method using pressure support ventilation (PSV) to determine whether a patient would tolerate IMV interruption. This ventilation mode consists of a pressure support of 7 cm H_2_O (the minimum level to overcome circuit resistance), positive end-expiratory pressure (PEEP) of 5-8 cm H_2_O (nearest to physiological values), and inspired oxygen fraction (FiO_2_) ≤ 40%. This trial lasts 30-120 min and is helpful in identifying patients who are able to maintain spontaneous breathing^[8]^.

Following endotracheal tube and artificial ventilation removal, respiratory support should be provided with oxygen to ensure arterial oxygen saturation (SaO_2_) close to physiological levels (95%). Oxygen therapy can be offered using a nasal catheter, nebulization mask, or Venturi system^[6]^.

In this study, we investigated the effects of different PEEP levels applied during SBT on oxygenation indices in patients undergoing CABG.

## METHODS

We performed a randomized clinical trial with 78 patients undergoing CABG between August 2013 and March 2014 who were admitted to the Cardiovascular Intensive Care Unit (ICU) at Hospital Universitário da Universidade Federal do Maranhão, in São Luís, Maranhão, Brazil. We excluded patients with chronic obstructive pulmonary disease and those undergoing emergency, off-pump, or combined surgeries. We excluded patients who required surgical reintervention or noninvasive ventilation during the first 6 h after extubation.

Before surgery, patients received explanations and information about the research. After surgery, data were collected from physiotherapy evaluation forms and medical records. All data were registered in a form that captured preoperative, intraoperative, and postoperative periods. All included patients underwent general anesthesia and median sternotomy.

After ICU admission, mechanical ventilation was applied using an Evita 2 Dura (Dräger Medical, Lübeck, Germany). Patients were ventilated in volume-controlled mode, according to the routine protocol, with the following settings: a tidal volume of 6–8 mL/kg, respiratory rate of 14 bpm, inspiratory flow of 8-10 times the minute volume, inspiratory time of 1 s, and inspired oxygen fraction of 40%.

During the preoperative period, patients were randomized into groups by simple draw, and this information was shared with the ICU care providers. SBT was initiated once the following clinical conditions were met: hemodynamic stability, absence of bleeding or minimal bleeding, absence of vasopressor use or low and stable doses of vasopressors, Glasgow Coma Scale ≥ 10 and strong respiratory drive.

The spontaneous breathing trial was administered using pressure support ventilation (support pressure 7 cm H_2_O and FiO_2_ 30%). The sample was divided into three groups: Group A, PEEP = 5 cm H_2_O; Group B, PEEP = 8 cm H_2_O; and Group C, PEEP = 10 cm H_2_O. Extubation was performed after 30–120 min with no destabilization signs. Following extubation, all patients received additional oxygen support by Venturi mask (Galemed Corporation, Wu-Jia, Taiwan) with an FiO_2_ of 31% to ensure arterial oxygen saturation close to physiological levels (around 95%).

Arterial blood samples were collected before extubation and at 1, 3, and 6 h after mechanical ventilation withdrawal. Samples were processed by an ABL 800 FLEX blood gas analyzer (Radiometer, Bronshoj, Denmark), according to the routine protocol. We then identified the arterial oxygen partial pressure (PaO_2_) and PaO_2_/FiO_2_ ratio.

Following the first arterial blood gas analysis after extubation, oxygen support was adjusted according to the necessary inspired oxygen fraction (FiO_2N_). To estimate the ideal arterial oxygen partial pressure (PaO_2I_) for each patient, we used the following equation to account for age and supine position: PaO_2I_ = 109 − (0.43 × age)^[9]^.

The inspired oxygen fraction provided following extubation was calculated according to the following formula: FiO_2N_ = FiO_2K_ × PaO_2I_/PaO_2K_, in which FiO_2N_ = the inspired oxygen fraction necessary after extubation, FiO_2K_ = the inspired oxygen fraction applied at the moment of arterial blood sample collection, PaO_2I_ = the ideal arterial oxygen partial pressure, and PaO_2K_ = the arterial oxygen partial pressure as measured by the last arterial blood gas. Oxygen was administered by Venturi mask using the following criteria:

FiO_2N_ <21%: room air;

FiO_2N_ = 21%–24%: blue connector, FiO_2_ 24%, O_2_ flow 4 lpm;

FiO_2N_ = 24.1%–28%: yellow connector, FiO_2_ 28%, O_2_ flow 4 lpm;

FiO_2N_ = 28.1%–31%: white connector, FiO_2_ 31%, O_2_ flow 4 lpm;

FiO_2N_ 31.1%–35%: green connector, FiO_2_ 35%, O_2_ flow 6 lpm;

FiO_2N_ 35.1%–40%: red connector, FiO_2_ 40%, O_2_ flow 8 lpm;

FiO_2N_ >40%: orange connector, FiO_2_ 50%, O_2_ flow 12 lpm.

When noninvasive ventilation (NIV) was required following extubation, it was applied, as per the routine protocol, according to the individual's needs. It is noteworthy that patients who used NIV during the first 6 h after extubation were excluded.

Ethical approval was obtained from the local Ethics Committee (protocol Nº. 327.798), as required by Resolution 466/12 of the National Health Council. All patients provided written informed consent.

Data were evaluated using the Stata/SE statistical software version 11.1 (StataCorp, College Station, TX, USA). To test normality, we used the Shapiro–Wilk test. Quantitative variables were described as means and standard deviations, and differences were determined using the Student's t, ANOVA, or Kruskal–Wallis test, depending on normality. Qualitative variables were expressed as proportions and tested by G-test and William's correction. Results were considered statistically significant when *P* value was <0.05.

## RESULTS

**Fig. 1:**
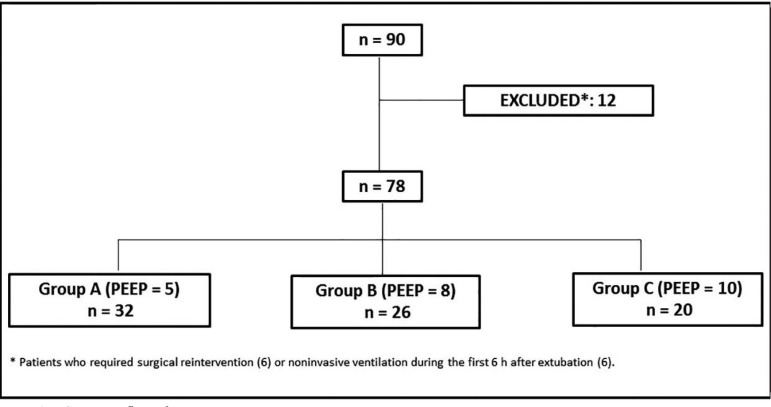
Consort flow diagram.

Ninety patients were randomized and underwent CABG during the study period. Of these, twelve (four of each group) were excluded because of postoperative surgical reintervention (6) and noninvasive ventilation use during the first 6 h after extubation (6) (Figure 1). Therefore, the final sample included 78 patients, who were predominantly male (69.3%) and from the countryside (53.8%), with a mean age of 61.7±8.6 years and body mass index of 26.1±3.7 kg/m^2^. Groups did not differ significantly with regard to demographic, clinical, or surgical variables, as seen in Tables 1 and 2.

**Table 1:** Demographic and clinical data for patients undergoing CABG.

Variables	Group A (n=32)	Group B (n=26)	Group C (n=20)	TOTAL(%)	*P*
Gender					0.49^a^
Male	26	16	12	54 (69.3)	
Female	6	10	8	24 (30.7)	
Age (years)	61.3±9.4	60.3±6.7	64.2±10	61.7±8.6	0.64^b^
Origin					0.15^a^
Capital	20	12	4	36 (46.2)	
Countryside	12	14	16	42 (53.8)	
BMI (kg/m^2^)	25.6±3.7	27.4±4.5	25.3±2.4	26.1±3.7	0.55^b^
Comorbidities					
Hypertension	20	18	12	50 (64.1)	0.81^a^
Diabetes mellitus	12	12	6	30 (38.4)	0.52^a^
Dyslipidemia	8	12	4	24 (30.7)	0.40^a^
Smoking	6	10	6	22 (28.2)	0.49^a^
Myocardial infarction	6	2	2	10 (12.8)	0.69^a^

BMI=body mass index.

a G test.

b Kruskal-Wallis test

**Table 2:** Surgical data for patients undergoing CABG.

Variables	Group A (n = 32)	Group B (n = 26)	Group C (n = 20)	*P*
Number of bypasses	3 (2.75;3)	2 (2;3)	3 (2;3)	0.22^a^
Number of drainage tubes	2 (2;2)	2 (2;2)	2 (2;2)	0.56^a^
Pump time (min)	83.4 ± 21.7	67.3 ± 25.3	89.4 ± 23.5	0.08^b^
Aortic clamp time (min)	60.9 ± 19.2	48.4 ± 20.2	61.5 ± 17	0.15^b^
Surgery time (min)	220.9 ± 25.3	230.5 ± 62.1	257.6 ± 64.9	0.22^a^

Data shown as the mean±standard deviation or median (1^st^ quartile; 3^rd^ quartile).

a ANOVA.

b Kruskal-Wallis test

The mean mechanical ventilation duration was 12.8±6.9 h. Patients in Group A (PEEP 5 cm H_2_O) were ventilated for 13.6±8.1 h, whereas those in Group B (PEEP 8 cm H_2_O) were ventilated for 11.7±6 h and those in Group C (PEEP 10 cm H_2_O) were ventilated for 13.2±4.8 h (*P*=0.69). There were no differences in mean gas exchange values (PaO_2_/FiO_2_) between groups at 1, 3, and 6 h after extubation (Table 3).

**Table 3:** Comparison of gas exchange mean (mmHg) between the three groups of patients undergoing CABG.

Groups/times	1^st^ hour	3^rd^ hour	6^th^ hour
Group A	320.5±65	347.7±75.9	333.1±67.9
Group B	326.9±84.1	332.5±97.3	343.5±118.5
Group C	308.3±49.9	313.3±56.9	311.5±80.3
*P*	0.92	0.64	0.77

Data shown as the mean±standard deviation. Kruskal-Wallis test.

Mean arterial oxygen saturation and inspired oxygen fraction did not differ between groups at 1, 3, and 6 h after extubation (Tables 4 and 5).

**Table 4:** Comparison of arterial oxygen saturation (%) between the three groups of patients undergoing CABG.

Groups/times	1^st^ hour	3^rd^ hour	6^th^ hour
Group A	97.6±0.9	97.0±0.9	96.8±0.8
Group lB	97.2±1.9	96.8±1.9	96.9±1.2
Group C	97.5±1.2	96.7±1.4	96.8±1.1
*P*	0.84	0.86	0.65

Data shown as the mean±standard deviation. Kruskal-Wallis test.

**Table 5:** Comparison of inspired oxygen fraction (%) applied after extubation between the three groups of patients undergoing CABG.

Groups/times	1^st^ hour	3^rd^ hour	6^th^ hour
Group A	26±5	27±6	27±5
Group B	27±5	28±7	28±8
Group C	27±5	27±6	27±7
*P*	0.61	0.70	0.77

Data shown as the mean ± standard deviation. Kruskal-Wallis test

## DISCUSSION

Gas exchange impairment is a significant complication during the CABG postoperative period^[10]^. In thoracic surgeries, these changes may be related to intraoperative procedures, such as mechanical ventilation with low volumes and PEEP, pain, and thoracotomy (which alters chest wall compliance)^[11,12]^. Therefore, we chose to evaluate oxygenation indices after extubation, because they properly reflect changes in pulmonary function following on-pump surgery^[13]^.

To reopen collapsed lung units and improve arterial oxygenation following thoracic surgery, different PEEP levels have been proposed^[14]^. Dongelmans et al.^[15]^, who compared high versus physiological PEEP (10 vs. 5 cm H_2_O) after CABG, showed that the highest PEEP levels improve oxygenation and lung compliance but are associated with increased mechanical ventilation duration. In their randomized clinical trial of 136 patients undergoing CABG who were mechanically ventilated at 5, 8, or 10 cm H_2_O of PEEP, Borges et al.^[16]^ showed that the highest PEEP levels may increase respiratory mechanics and provide better oxygenation indices in the immediate postoperative period.

Our hypothesis that application of higher PEEP levels throughout SBT would improve oxygenation after extubation was not supported by our measurements during the first 6 h after extubation. The results were consistent with those measured in the randomized clinical trial by Marvel et al.^[17]^, in which patients undergoing CABG and ventilation with PEEP of 0, 5, or 10 cm H_2_O did not experience a sustained arterial oxygenation benefit from higher PEEP levels.

A question that arose during our research was what PEEP level would be considered physiological to avoid alveolar collapse while performing SBT, given that the "expiratory delay function" of the glottis (which serves as an organic PEEP mechanism to prevent or minimize alveolar collapse) is removed during artificial ventilation^[18]^? During mechanical ventilation of adult patients, PEEP is generally set to 3–5 cm H_2_O, as this is considered physiological^[19]^. However, our study provided some evidence that levels between 5 and 8 cm H_2_O, possibly up to 10 cm H_2_O, may more closely mimic normal respiratory physiology for such patients.

The knowledge of physical therapy was found to be generally applied across the entire treatment process^[20]^. Physical therapists play an important role in conducting patient-screening protocols for mechanical ventilation weaning^[21,22]^. Our research emphasizes identification of optimal variables during weaning as fundamental to this process so as to minimize patient complications.

## CONCLUSION

In this sample of patients undergoing CABG, the use of different PEEP levels before extubation did not affect gas exchange or oxygen therapy utilization in the first 6 h after endotracheal tube removal.

**Table:** 

**Authors’ roles & responsibilities**	
ROL	Analysis and/or interpretation of data; study design; implementation of projects and/or experiments; manuscript writing or critical review of its content
DLB	Analysis and/or interpretation of data; statistical analysis; final approval of the manuscript; study design; implementation of projects and/or experiments; manuscript writing or critical review of its content
MAGC	Conduct of operations and/or experiments
TEPB	Conduct of operations and/or experiments
MGBS	Conduct of operations and/or experiments
FASS	Conduct of operations and/or experiments
MOS	Conduct of operations and/or experiments
JGMP	Analysis and/or interpretation of data; manuscript writing or critical review of its content
